# Randomised controlled trials of group interventions with an individual level comparator: are appropriate statistical methods being used?

**DOI:** 10.1186/1745-6215-16-S2-P125

**Published:** 2015-11-16

**Authors:** Lucy Bradshaw, Alan Montgomery

**Affiliations:** 1 Nottingham Clinical Trials Unit, University of Nottingham, Nottingham, UK

## Background

Differential clustering can occur in trials when a group-based intervention is compared with a control arm in which participants are treated individually. Recommendations for the design and analysis of this type of trial have been published. This review aimed to investigate use of these methods in trials of group interventions with an individual level comparator.

## Methods

Protocols of randomised trials published in Trials journal in 2013 and 2014 were reviewed. Data were extracted by one reviewer on randomisation, the group intervention, the sample size calculation and the planned statistical analysis.

## Results

A total of 738 protocols were published in Trials in 2013/2014 and 37 (5%) were included in the review. Group interventions included psychological therapies, lifestyle interventions, parenting programmes and exercise interventions. Sample sizes ranged from 35 to 796, median 112 and all used a 1:1 allocation ratio. Eight trials (22%, 95% CI 9% to 38%) recognised the potential for differential clustering between treatment arms. Three trials inflated the sample size of the trial, three trials planned to account for the differential clustering between the groups in the primary analysis and three trials planned to explore the effect of differential clustering in a planned sensitivity analysis.

## Conclusions

Only a small number of protocols identified the potential for differential clustering and accounted for it in the design or planned analysis, despite the availability of appropriate methods to do so. Results from trials that fail to recognise such clustering require careful interpretation.

